# Anti-Thrombin, Anti-Adhesive, Anti-Migratory, and Anti-Proliferative Activities of Sulfated Galactans from the Tropical Green Seaweed, *Udotea flabellum*

**DOI:** 10.3390/md17010005

**Published:** 2018-12-21

**Authors:** Maxsuell Lucas Mendes Marques, Fernando Bastos Presa, Rony Lucas Silva Viana, Mariana Santana Santos Pereira Costa, Monica Oliveira Rocha Amorim, Daniel Lima Bellan, Monique Gabriela Chagas Faustino Alves, Leandro Silva Costa, Edvaldo Silva Trindade, Hugo Alexandre Oliveira Rocha

**Affiliations:** 1 Laboratório de Biotecnologia de Polímeros Naturais—BIOPOL, Departamento de Bioquímica, Universidade Federal do Rio Grande do Norte, Natal 59078-970, Brazil; maxsuell_lucas@hotmail.com (M.L.M.M.); fernandobpresa@gmail.com (F.B.P.); rony_lucas@hotmail.com (R.L.S.V.); monicaamorimrn@hotmail.com (M.O.R.A.); monique.gabi@gmail.com (M.G.C.F.A.); 2 Programa de Pós-Graduação em Ciências da Saúde, Universidade Federal do Rio Grande do Norte, Natal 59078-970, Brazil; 3 Instituto Federal de Educação, Ciência, e Tecnologia do Rio Grande do Norte (IFRN), Canguaretama-RN 59500-000, Brazil; mariana.costa@ifrn.edu.br (M.S.S.P.C.); leandro-silva-costa@hotmail.com (L.S.C.); 4 Departamento de Biologia Celular, Universidade Federal do Paraná, Curitiba 80060-000, Brazil; daniellimabellan@gmail.com (D.L.B.); edstrindad@gmail.com (E.S.T.)

**Keywords:** tropical marine seaweeds, sulfated polysaccharides, anti-thrombin, antitumor

## Abstract

In this study, sulfated polysaccharide-rich extracts were isolated from 22 tropical seaweeds (4 red, 11 brown, and 7 green) found in northeastern Brazil, and evaluated for the role of anticoagulant agents. Fifteen of the extracts showed anticoagulant activity, including all the extracts from green seaweeds. *Udotea* *flabellum* (a green seaweed) extract was the most potent, requiring an amount of only 3 µg to double the plasma coagulation time in the activated partial thromboplastin time test. A similar result was obtained with 1 µg of heparin. Two sulfated homogalactans with anticoagulant activity, F-I (130 kDa) and F-II (75 kDa), were isolated from this extract using several bio-guided purification steps. Their anticoagulant activity, as well as properties related to antitumor activity (anti-proliferative, anti-adhesive, and anti-migratory), were accessed. Their anticoagulant activities were close to that of heparin. We found that F-I and F-II (0.5–10 μg/mL) were not able to directly inhibit thrombin. In the presence of anti-thrombin, F-I (0.5 μg/mL) was more effective than heparin (0.5 μg/mL) in inhibiting thrombin, while F-II showed similar effects as heparin. F-I and F-II also inhibited B16-F10 (murine melanoma cells) adhesion, migration, and proliferation on a fibronectin-coated surface, but not on laminin- or collagen I-coated surfaces. Except for the antiproliferative activity, the other effects of F-I and F-II were eliminated upon their desulfation (~50%), indicating that the degree of sulfation is not as important for F-I and F-II anti-proliferative activity as the sulfation position. Taken together, the results provide strong evidence for the potential utility of sulfated galactans from *U.* *flabellum*, making these compounds an interesting option for future investigations that aim to design new anticoagulant/antitumor agents.

## 1. Introduction

Cancer is a broad term used to represent a class of over 100 different types of diseases that share specific features, including disordered proliferation of abnormal cells with invasive potential. Trachea, bronchus, lung, colon, rectum and breast cancers were some of the leading causes of death in the Western world in 2016 [1]. Evidence has been accumulated showing that tumor progression is accompanied by a hypercoagulability state resulting from excessive blood coagulation activation [2]. In fact, cancer-associated thrombosis is the second leading cause of death in cancer patients, after death from cancer itself. For example, the risk of a venous thromboembolism is four- to seven-fold higher in patients with cancer than in those without cancer. The clinical presentation of cancer-associated thrombosis includes deep vein thrombosis, pulmonary embolism, chronic disseminated intravascular coagulation associated with non-bacterial thrombotic endocarditis, and arterial thrombosis. In some reports, secondary thrombotic events related to chemotherapy or central venous catheterization are included in the description of cancer-associated thrombosis [3].

Many cancer cells have abnormally high constitutive levels of tissue factor (TF) and elicit procoagulant activities by an expression of TF. In addition, endothelial cells (EC) are induced by inflammatory cytokines produced by tumor cells, which causes EC to produce TF. Monocytes/macrophages can also be induced by tumor cells to release TF [4]. The data indicate that tumors induce the formation of TF/factor VIIa complex, which in turn activates factor X and, consequently, thrombin (factor II), which leads to thrombus formation (cancer-associated thrombosis). It has been reported that, in addition to coagulation, TF, activated factor X (FXa) and thrombin (IIa) are involved in the proliferation and migration of tumor cells, as well as in the induction of angiogenesis by tumors [5].

Therapeutic anticoagulation, without interruption, is the most critical component in the management of patients with cancer-associated thrombosis. However, warfarin, oral anticoagulant, may be ineffective in these patients and has been associated with venous limb gangrene in patients with cancer and thrombosis [6]. Rivaroxaban, a factor Xa inhibitor, has also been associated with venous limb gangrene in patients with acute cancer-associated thrombosis [7]. The main known anticoagulant drug is the sulfated polysaccharide (SP) named heparin [8]. It reduces the risk of venous thromboembolism in cancer-associated thrombosis patients because it strongly inhibits the action on blood coagulation proteases. In addition, it inhibits tumorigenic actions induced by IIa and FXa, conferring a survival benefit in cancer patients and the regression of primary tumors [9]. However, the use of heparin can cause side effects such as the development of thrombocytopenia, arterial embolism, bleeding complications, and even death of the patient [10]. In addition, the problem of heparin deliberate contamination with super sulfated chondroitin can induce death of the patient [11]. 

Many sulfated seaweed polysaccharides have anticoagulant activities [12,13]. In fact, they have high potential as preventive and therapeutic agents against several diseases, for their anticancer, anti-inflammatory, antioxidant, antibacterial and anticoagulant properties [13]. Polysaccharides from seaweeds are only partly explored, due to their great diversity, and metabolite complexity of seaweeds offers a unique and exclusive source of active polysaccharides. It is clear that anticoagulant activity is not exclusively dependent on the sulfate content of SP. SP activity depends on its negative charge, specifically, on the distribution of this charge throughout the molecule; this influences how SP interacts with clotting factors and natural inhibitors, such as antithrombin (AT) and heparin cofactor II (HCII), which determines whether or not blood anticoagulation is promoted [14]. 

Heparin can also be achieved through mechanisms independent of its anticoagulant action, such as interfering with proliferation, adhesion and migration of tumor cells [9]. SPs from seaweeds have already proved to have these important properties and to show promise as bioactive products and biomaterials with a wide range of applications, including antitumor [13]. In addition, a feature of seaweed SP is that each type of seaweed synthesizes at least one SP that is unique and therefore may be one that is more suitable than that currently used for a certain application [13]. Thus, each new purified SP presents great potential to be explored. Therefore, our goal was to obtain an anticoagulant SP from seaweed that possesses also other important activities that can be used against tumor cells. Thus, in this paper, the SP-rich extracts obtained from 22 tropical seaweeds found in northeastern Brazil were evaluated as anticoagulant agents. We chose the most potent extract, and its polysaccharides were purified by a bioguided process. Two sulfated polysaccharides were obtained and analyzed as anticoagulant, thrombin inhibiting, anti-proliferative, cytotoxic, anti-migratory and anti-adhesive agents. 

## 2. Results and Discussion

### 2.1. Evaluation of Anticoagulant Activity of SP-Rich Extracts from Tropical Seaweeds

SP-rich extracts were isolated from the tropical seaweeds described in Section 3, and their anticoagulant activities were determined using the activated partial thromboplastin time (APTT) method. The results are shown in Table 1; more than 50% of the extracts succeeded in altering the plasma coagulation time. However, the activity (in terms of the amount (µg) required to double APTT compared with saline control) was not the same for all the extracts. For example, the extract of *Gracilaria birdiae* was effective only at a high amount of 100 µg. Six extracts, all from brown seaweeds, showed stipulated activity only at quantities above 10 µg. In contrast, all extracts obtained from green seaweeds exhibited good anticoagulant activity. In particular, *U. flabellum* extract doubled the plasma coagulation time at a quantity as low as 3 µg.

The SPs of the green seaweed *U. flabellum* had the highest anticoagulant activity. This was not surprising given that Arata et al. [15] had already reported anticoagulant activity of the SPs from this seaweed. However, the anticoagulant activity of *Udotea* SP presented in their study was much lower than that observed in our study. The structures of seaweed SPs can vary depending on environmental factors, as shown by Siddhanta et al. [16]. These authors showed that the yield, monosaccharide composition, and amount of SP from the green seaweed *Ulva fasciata* varied seasonally. It is possible that the difference between the anticoagulant activity reported by Arata et al. [15] and us could be explained by such seasonal variations. As *U. flabellum* SP showed the most potent activity in APTT anticoagulant tests, we chose this SP for subsequent steps.

### 2.2. Obtaining the SP-Rich Fractions from the Seaweed U. flabellum

In order to identify the anticoagulant SP within the *Udotea* extract, we fractionated the extract by adding increasing volumes of acetone, as described in Section 3. After adding two volumes of acetone, no further precipitates were formed. Thus, six SP-rich fractions from *U. flabellum* were obtained when 0.3, 0.5, 0.6, 0.7, 1.0, and 2.0 volumes of acetone were used. These fractions were named according to the acetone volume used to obtain them, i.e., UF-0.3, UF-0.5, UF -0.6, UF -0.7, UF-1.0, and UF-2.0, respectively. 

The fractionation yield and the anticoagulant activity of these fractions are shown in Table 2. Fraction UF-0.5 yielded the highest quantity (~34%), followed by fraction UF-2.0 (~20%). On the other hand, the lowest yield was obtained for fraction UF-0.3 (~5%). Recently, using this same fractionation protocol, Presa et al. [17] also obtained six SP-rich fractions from *U. flabellum*, with very similar yields to those observed in Table 2. *U. flabellum* belongs to order Bryopsidales. Two other seaweeds of this order, *Codium isthmocladum* [18] and *Caulerpa cupressoides* [19], were also subjected to acetone fractionation, resulting in five and four SP-rich fractions, respectively. These results indicate that this class of seaweed synthesizes more than one type of SP.

### 2.3. Purification of SPs from the UF-0.5 Fraction

Fraction UF-0.5 yielded the highest quantity of precipitate with acetone. It was also the fraction that presented the highest anticoagulant activity (Table 2). Therefore, this fraction was chosen to purify the SPs with anticoagulant activity. SPs from UF-0.5 were purified according to their molecular mass as described above. In total, five SPs were obtained, which were named F-I, F-II, F-III, F-IV, and F-V. The results of the chemical analysis and the APTT of these SPs are summarized in Table 3.

Xylose was found to be one of the monosaccharides in the samples because 3-linked β-d-xylans replaces cellulose in *U. flabellum* [20]. Mannose and glucose have been previously described as components of SP-rich extracts obtained from *Udotea* seaweed [15]. However, the previously reported amounts of these monosaccharides are much lower than what we report. This difference can be explained by seasonal differences, as well as by the extraction method, as we used a different extraction method from those used in the previous papers. Several authors have reported that different methods of extraction of SP yield different monosaccharide compositions [13]. Galactose was the predominant monosaccharide in all samples, with F-I and F-II comprising almost exclusively this monosaccharide. This agrees with data in the literature. Although there is not much data on the structure of SPs from order Bryopsidales, the main SPs described in these seaweeds are sulfated galactans [18,19,21,22,23].

All these SPs showed a high anticoagulant activity, especially F-I, F-II, and F-IV. However, F-III, F-IV, and F-V showed low amounts of sulfate and polysaccharide, although they were highly contaminated by proteins in comparison with F-I and F-II. Similar to heparin, only 1 µg was required to double the coagulation time for these two SPs.

**Figure 1:**
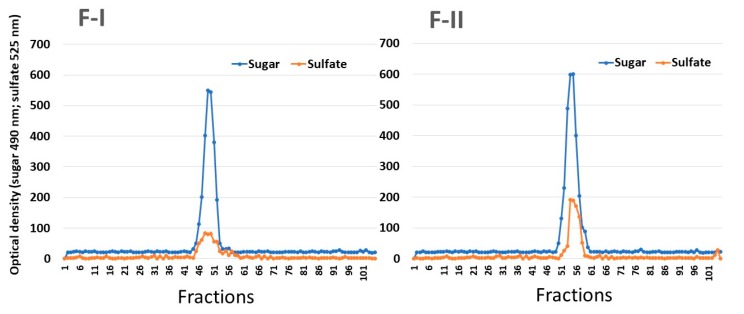
Gel permeation chromatography of SPs from *U. flabellum*. F-I and F-II were introduced into a Sephadex G-100 column (140 × 1 cm). The column was eluted with 0.2 M acetic acid, and 1 mL fractions were collected, which were then analyzed for the presence of sugars by the Dubois method and metachromasia, as described in Section 3.

In order to verify whether F-I and F-II consist of single compounds, they were subjected to gel filtration chromatography. As shown in Figure 1, F-I and FI-II produced only one peak in each chromatogram, indicating that these SPs consist of only one polysaccharide. In addition, it was possible to determine the apparent molecular weight of the samples (130 kDa and 75 kDa for F-I and F-II, respectively).

### 2.4. Inhibitory Effect of F-I and F-II on Thrombin Activity (Anti-Thrombin (AT) Activity)

Table 3 shows the high anticoagulant activity of F-I and F-II. In general, according to the existing literature, the anticoagulant mechanism-of-action of SPs from seaweed is based on the inhibition of thrombin via different mechanisms. Thus, to identify the anticoagulant mechanism of F-I and F-II, their inhibitory effects on thrombin were evaluated.

In the direct thrombin inhibition assay, the polysaccharides were tested at concentrations not exceeding 10 μg/mL, because above this concentration, a precipitate was formed. The data show that F-I and F-II (0.5 to 10 μg/mL) did not inhibit thrombin when incubated in the absence of AT or HCII. The anticoagulant SPs from green seaweed act via AT and HCII, as described for the SPs of green seaweeds *Codium adhaerence* and *Codium divaricatum* [24]. Alternatively, they may act via only HCII, as in the case of SP from green seaweeds *Caulerpa brachypus* and *Caulerpa okamurai* [24]. Lastly, they can act by both activating serpins and acting directly on thrombin, as the case of SPs from *Ulva conglobata* [25].

**Figure 2:**
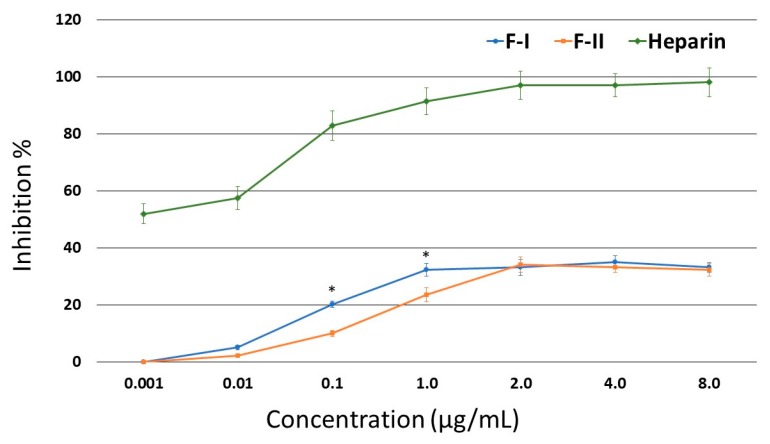
Inhibition of thrombin activity by HC-II in the presence of F-I or F-II. * *p* < 0.05 (F-I vs. F-II).

Thus, the inhibitory effects of F-I and F-II on thrombin were assessed in the presence of HCII. The purified SPs were able to inhibit thrombin (Figure 2). At lower concentrations, F-I was more active than F-II. However, neither of the two compounds showed an activity greater than 35%.

**Figure 3:**
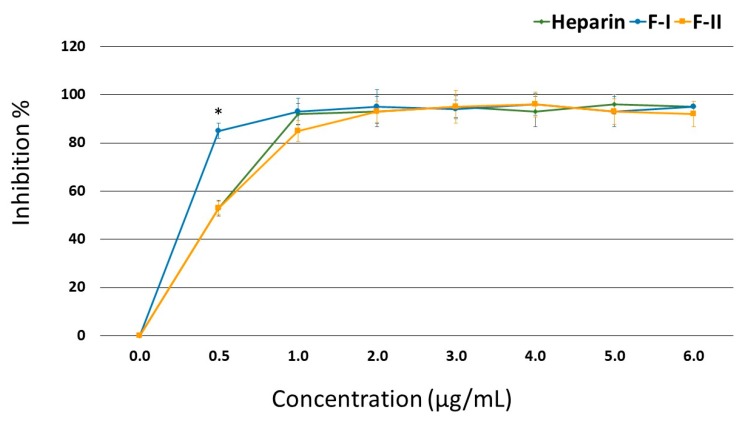
Inhibition of thrombin activity by anti-thrombin (AT) in the presence of F-I or F-II. * *p* < 0.05.

Figure 3 shows that in the presence of AT, F-II inhibited the thrombin activity just as efficiently as heparin; starting from 1.0 μg/mL, this effect reached approximately 93% inhibition. In the case of F-I, a concentration of 0.5 μg/mL showed a greater inhibitory effect than heparin (85.3% vs. 53.4% inhibition, respectively), but its inhibitory effect became similar to that of heparin from 1.0 μg/mL onwards.

The high anticoagulant activity observed in the APTT test was mainly due to the inhibition of thrombin by AT. This molecule also inhibits other coagulation factors. Thus, the possibility that these factors are inhibited by F-I and F-II cannot be discounted, and we intend to evaluate this issue in the future.

Our findings demonstrate the potential of F-I and F-II as anticoagulant agents, as they primarily inhibit AT. We intend to perform in vivo studies in future to evaluate the anticoagulant activity of F-I and F-II, as well as to determine whether or not these compounds exert any of the collateral effects exhibited by heparin.

In addition, many studies have indicated the importance of anticoagulants in the treatment of different types of tumors. The data indicate that tumors induce the formation of tissue factor/factor VIIa complex, which, in turn, activates factor X, and consequently, thrombin, which induces thrombus formation [26,27]. However, the release of heparin can also be achieved through mechanisms independent of its anticoagulant action, such as those interfering with proliferation, adhesion, and migration of tumor cells [9,28]. Therefore, we also observed the effect of F-I and F-II on the proliferation, migration, and adhesion of B16-F10 melanoma cells. This cell line was chosen because it is highly aggressive, and it successfully forms tumors in mouse xenograft models. Moreover, several reports have indicated that blood coagulation activation has a key role in melanoma progression [29].

### 2.5. Effect of Sulfated Polysaccharides on Cell Viability and Cell Proliferation

**Figure 4:**
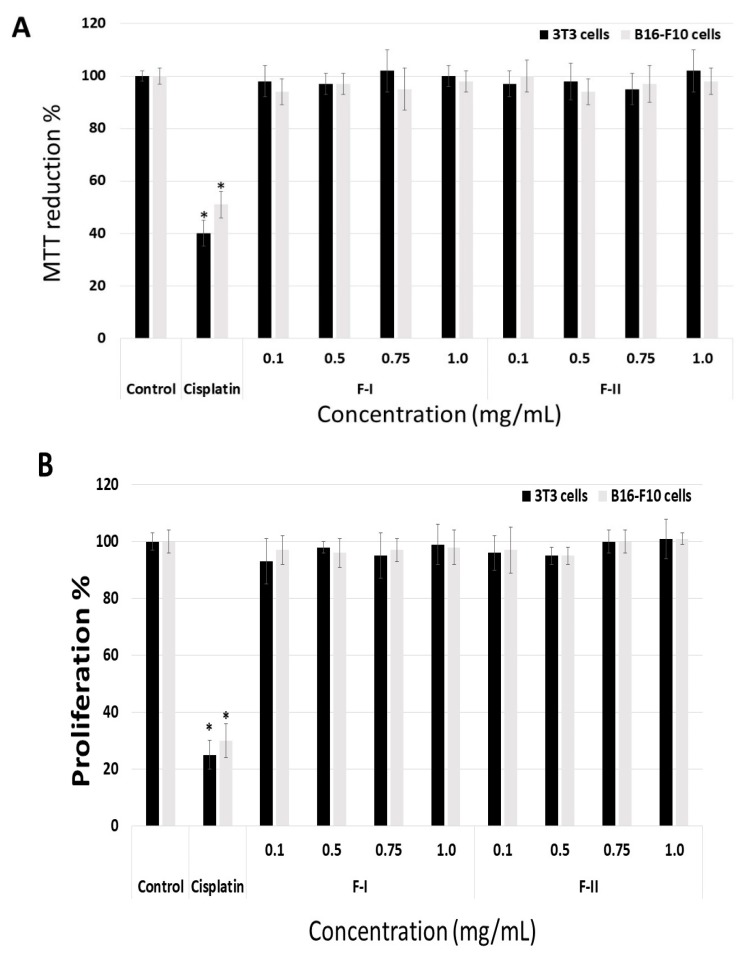
Effect of different concentrations (mg/mL) of F-I or F-II on the ability of fibroblast (3T3) cells to reduce MTT (3-(4,5-dimethylthiazol-2-yl)-2,5-diphenyltetrazolium bromide)) (**A**) and incorporate BrdU (5-Bromo-2-Deoxyuridine) (**B**) The cells were cultured with different concentrations of F-I or F-II (from 0.1 to 1.0 mg/mL) for 24 h, and subsequently subjected to MTT test or BrdU test. Control (negative control) consisted of only the culture medium with fetal bovine serum. Positive control comprised culture medium with fetal bovine serum and cisplatin (0.005 µg/mL). * *p* < 0.001 vs. control.

Initially, we evaluated whether F-I and F-II were toxic to B16-F10 tumor cells and 3T3 fibroblasts. Thus, the cells were exposed to polysaccharides (from 0.1 to 1.0 mg/mL) for 24 h, and afterwards they were evaluated by MTT (3-(4,5-dimethylthiazol-2-yl)-2,5-diphenyltetrazolium bromide)) and BrdU (5-Bromo-2-Deoxyuridine) tests. As seen in Figure 4A, the polysaccharides failed to have any effect on the ability of 3T3 or B16-F10 cells to reduce MTT. In contrast, treatment with cisplatin (0.005 mg/mL), which was used as a positive control, caused 3T3 and B16-F10 cells to reduce 40% and 51% of the MTT molecules, respectively. Cisplatin is a well-known chemotherapeutic drug used for treatment of numerous human cancers [30]. In addition, even when used at low concentrations (0.005–0.010 mg/mL), cisplatin has an antiproliferative effect on cells in culture, as shown in other studies [17,30].

Figure 4B shows that the sulfated polysaccharides did not affect the proliferation of B16-F10 and 3T3 cells either, as the incorporation of BrdU (5-Bromo-2-Deoxyuridine) by the cells incubated in the presence of fractions was similar to that of the cells in the negative control group (cells exposed only to medium and fetal bovine serum).

### 2.6. Effect of Sulfated Polysaccharides on Cell Adhesion

**Figure 5:**
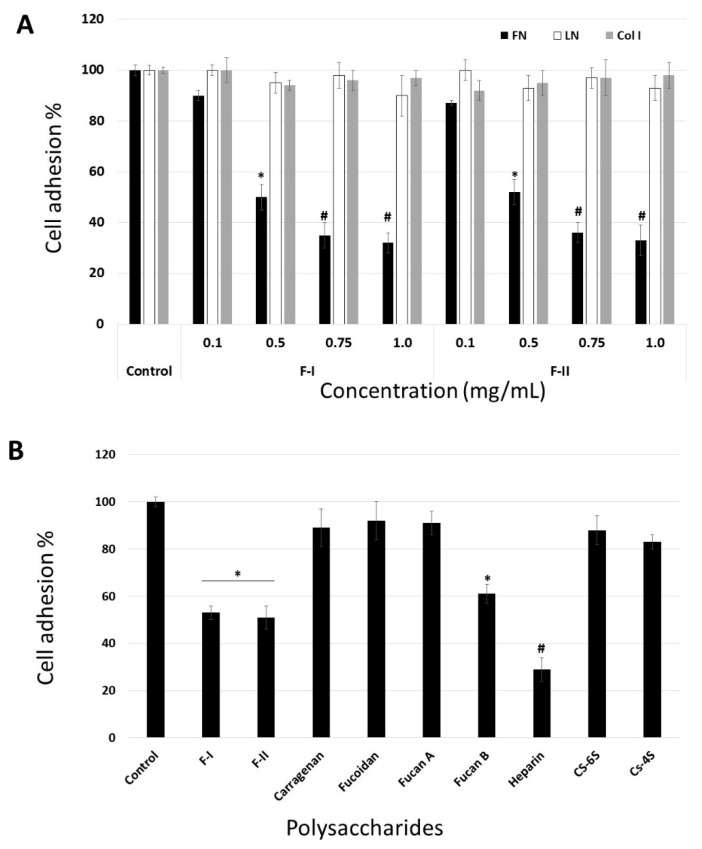
(**A**) Anti-adhesive effect of F-I and F-II on B16-F10 attachment to fibronectin. (**B**) Effects of different sulfated polysaccharides (0.5 mg/mL) on B16-F10 adhesion to fibronectin. Fuc B and Fuc A—fucan A and B from brown seaweed *Spatoglossum schroederi*, respectively; Hep—heparin; CS-6S—chondroitin-6-sulfate; CS-4S—chondroitin-4-sulfate; fucoidan—SP obtained from brown seaweed *Fucus vesiculosus*. * *p* < 0.05 vs. control. # *p* < 0.001 vs. control.

Tumor cells tend to migrate to the basement membranes, where they are known to adhere strongly. In fact, the invasion of tumor cells through basement membranes and adhesion to extracellular matrix proteins (laminin, fibronectin, vitronectin, etc.) are the crucial steps in metastasis. Therefore, drugs that inhibit cell adhesion should prevent the formation of metastasis [31]. Thus, we studied the ability of B16-F10 cells to interact with exogenous proteins, e.g., fibronectin (FN), laminin (LN), and type I collagen (Col I) in the presence of F-I or F-II (from 0.1 to 1.0 mg/mL), in a cell adhesion assay. The data are shown in Figure 5.

Figure 5A demonstrates that these effects of F-I and F-II are dose-dependent, reaching saturation at around 0.75 mg/mL. Evaluation of the viability of non-adherent cells, using the trypan blue exclusion assay, showed that the cells detached by F-I or F-II were perfectly viable. These data rule out the possibility that the anti-adhesive effect could be related to the anti-proliferative effect of F-I or F-II.

In order to analyze the effect of F-I and F-II structure on this inhibition, we compared the adhesion of B16-F10 cells to FN in the absence (control) or presence of or 0.5 mg/mL each of the following sulfated polysaccharides: F-I, F-II, heparin (Hep), chondroitin-4-sulfate (CS-4S), chondroitin-6-sulfate (CS-6S), and fucan B and fucan A from *S. schroederi* (Figure 5B). It is clear from Figure 5B that some sulfated polysaccharides inhibited the adhesion of B16-F10 cell to FN. Among the tested polysaccharides, heparin was the most potent anti-adhesive molecule, followed by F-I, F-II, and fucan B. This inhibitory effect does not seem to be solely dependent on sulfate content, as other sulfated polysaccharides such as fucan A did not exhibit any effect on cell attachment to FN. Furthermore, although F-I, F-II, and fucan B have sulfated galactose in their composition, the anti-adhesive effect of the sulfated polysaccharides was not dependent on the presence of sulfated galactose, as carrageenan, a sulfated homoglactan, did show any anti-adhesive effect.

Several sulfated polysaccharides have shown anti-adhesive properties in different systems [32,33,34,35,36]. With respect to SPs of green algae, such as *U. flabellum*, there is a report on another SP with anti-adhesive activity, an ulvan extracted from the green seaweed *Ulva* sp. [34]. This suggests that the ability to reduce cell adhesion to extracellular matrix components is a feature restricted only to a few sulfated polysaccharides [35], and that the biological activity of sulfated polysaccharides is unlikely to be merely a charge density effect.

**Figure 6:**
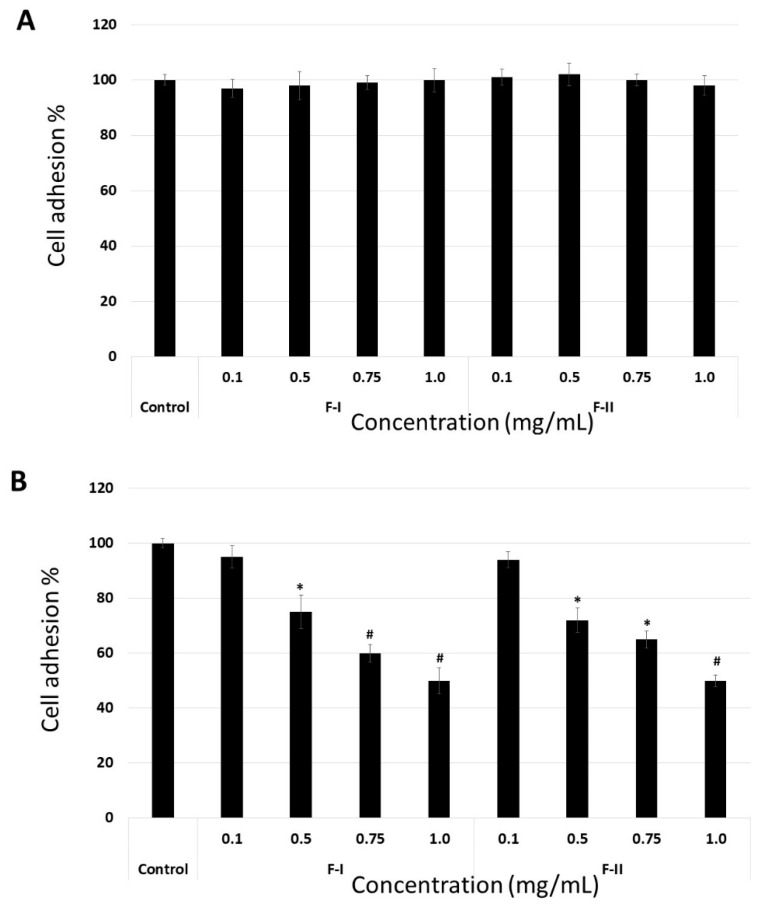
B16-F10 cell adhesion to fibronectin in modified adhesion assay. (**A**) Cells were exposed to F-I or F-II, washed, and subjected to cell adhesion assay. (**B**) The plates were coated with FN and F-I or F-II (0.1 to 1.0 mg/mL), and adhesion assay was carried out following the protocol described in Section 3. * *p* < 0.05 vs. control. # *p* < 0.001 vs. control.

The data indicate that the molecular target of F-I and F-II is FN. To confirm this, the adhesion assay was modified. In the first assay, the suspension cells were maintained at 4 °C in the presence of F-1 or F-II (0.1 to 1.0 mg/mL) for 1 h. Thereafter, they were washed with phosphate buffered saline (PBS), also at 4 °C, and subjected to the adhesion test as described above. In another assay, the plates were coated with FN, and thereafter, medium (100 µL) containing F-I or F-II (0.1 to 1.0 mg/mL) was added. These plates were left for 4 h under culture conditions (37 °C, 5% CO_2_). Subsequently, the plates were washed, and the cells were subjected to this adhesion assay using these plates. In Figure 6A, we can observe that F-I and F-II did not affect cell adhesion when the cells were exposed to them prior to the adhesion assay. This indicates that polysaccharides do not bind to the cell surface receptors that are involved in the process of cell adhesion. On the other hand, when FN was exposed to polysaccharides, they affected the adhesion of B16-F10 cells (Figure 6B). This indicates that the molecular target of F-I and F-II is FN.

To date, there are no data on whether SPs from green seaweed bind fibronectin. However, Liu et al. [36] showed that FN has at least four binding sites along its chain for fucans, two of which are also heparin-binding sites. Thus, we believe that at least one of these sites in FN is also the molecular target of the polysaccharides from *U. flabellum*.

### 2.7. The Effect of F-I and F-II on B16-F10 Migration on Laminin, Collagen I, and Fibronectin

Metastasis represents an advanced stage of malignancy and is the leading cause of cancer-related deaths. It is a multi-step process that includes cell migration, among others, and leads to the generation of secondary tumors [37]. Thus, we next evaluated the effect of F-I and F-II on B16-F10 migration.

**Figure 7:**
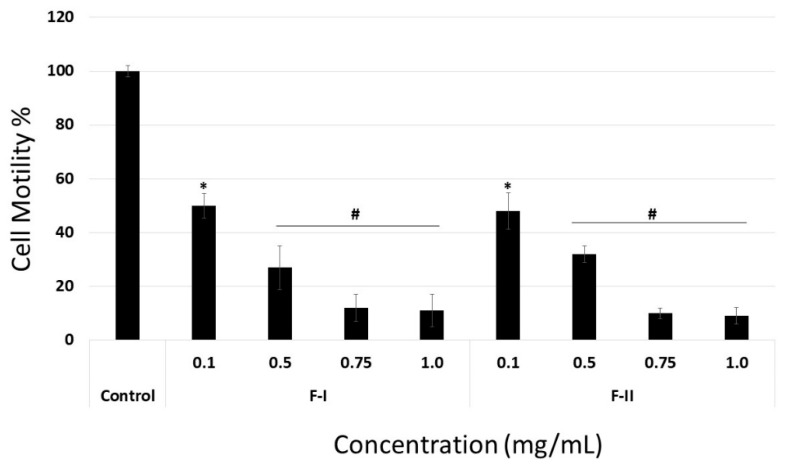
Motility on fibronectin of B16-F10 cell exposed to F-I or F-II. Cells on the other side of the membrane were stained with 1% toluidine blue and counted. Results are expressed as mean ± SD of three determinations. * *p* < 0.05 vs. control. # *p* < 0.001 vs. control.

When laminin or collagen I was the substrate, F-I and F-II did not affect cell migration at any of the tested concentrations (data not shown). In contrast, when fibronectin was the substrate, even the lowest tested samples concentration (0.1 mg/mL) inhibited cell migration by about 50% (Figure 7). In addition, the anti-migratory effect of F-I and F-II was dose-dependent, like the adhesion assay.

### 2.8. Antiproliferative Effect of F-I and F-II on B16-F10 Cells Coated onto Laminin, Collagen I, or Fibronectin

F-I and F-II did not inhibit the proliferation of B16-F10 cells when they were plated on plastic. On the other hand, they inhibited the adhesion and migration of B16-F10 cells when the substrate was Fn. Therefore, B16-F10 cells were seeded into wells coated with fibronectin (FN), collagen I, or laminin (LN). After 24 h, F-I or F-II (from 0.1 mg/mL to 1 mg/mL) was added and the cells were stimulated for growth.

**Figure 8:**
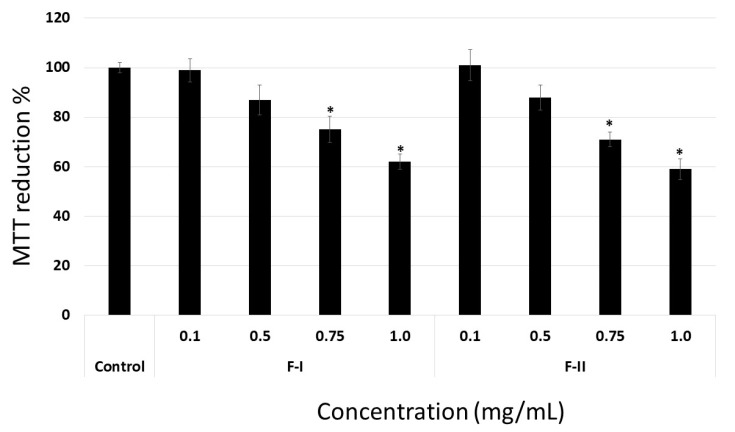
Anti-proliferative effect of F-I and F-II (from 0.1 to 1.0 mg/mL) on B16-F10 cells coated onto fibronectin. Data are presented as means ± standard deviation. * *p* < 0.05 vs. control.

When the cells were coated onto LN or collagen, F-I and F-II did not show any anti-proliferative activity (data not shown), even though they acted as an anti-proliferative molecule when FN was used as substrate for cell proliferation (Figure 8). Indeed, F-I and F-II (1.0 mg/mL) inhibit around 40% of B16-F10 cell proliferation, which is greater than the effect observed when plastic was used as a substrate. This percentage of inhibition did not increase significantly when F-I and F-II were evaluated at a higher concentration (1.5 and 2.0 mg/mL) (data not shown). Data indicate that the SP from *U. flabellum* binds extracellular matrix proteins, mainly FN, and acts as an anti-proliferative compound.

Other SPs also bind FN and inhibit cell proliferation, such as a fucan from *S. schroederi* [38], which inhibits Chinese ovarian cell (CHO-K1 cells) proliferation, and a fucoidan from *Ascophyllum nodosum* that is able to bind fibronectin and arrest the proliferation of MDA-MB-231 cells (adenocarcinoma from human breast) [36]. However, even at high concentrations (1.0 mg/mL), these two polysaccharides inhibited cell proliferation by less than 60%, which is close to the data observed for F-I and F-II. To the best of our knowledge, this is the first time a green seaweed SP has been shown to inhibit cell proliferation by binding to the extracellular matrix (specifically, to FN).

### 2.9. Properties of Desulfated F-I and F-II

It has been reported that SP bioactivities are closely related to several structural parameters, such as degree of sulfation and sulfation position [32]. In order to determine the role of sulfate groups in the activities of F-I and F-II, we used desulfated F-I and F-II (see Section 3) containing 7.0% and 7.5% of sulfate groups, respectively. Table 4 shows the data for the desulfated polysaccharides. The data indicated that none of the desulfated polysaccharides displayed anticoagulant, anti-adhesive, or anti-migratory activity. On the other hand, when cells were coated onto FN, the polysaccharides inhibit around 40% of B16-F10 cell proliferation. In other words, the degree of sulfation is not as important for F-I and F-II anti-proliferative activity as the sulfation position, whereas for other activities, it is important. These data corroborate with those presented by Haroun-Bouhedja et al. [39], where the authors showed that fucoidan lost its anticoagulant activity upon desulfation but retained its anti-proliferative activity.

Since the desulfation method used in this study removes the sulfate groups nonspecifically, a specific relationship between sulfate position and bioactivity could not be elucidated. Further investigation using more specific desulfation methods would help determine the role of sulfation position on F-I and F-II anti-proliferative activity.

Many authors correlate antitumor capacity of some drugs with cytotoxicity or loss of cell viability. However, such drugs might damage or kill normal and healthy cells. Therefore, recently, there is a search for compounds that do not alter the cellular viability, but rather cause cellular modifications that lead to antitumor effects, with minimum side effects [40,41]. Based on this, the results presented on this study reveal a promising agent against melanoma cells, which is a potential candidate for in vivo evaluation of its antitumor effect in future studies.

## 3. Materials and Methods

### 3.1. Materials

Heparin, N-Benzoyl-Phe-Val-Arg-p-nitroanilide hydrochloride chromogenic substrate, heparin cofactor II, monosaccharides, Sephadex G-100, and dextran standards were purchased from Sigma-Aldrich Co. (St. Louis, MO, USA). Cell culture medium components (F-12 medium), trypsin and newborn calf serum (FCS) were obtained from Cultilab (Campinas, SP, Brazil). l-Glutamine, sodium bicarbonate, sodium pyruvate, and phosphate buffered saline (PBS) were purchased from Invitrogen Corporation (Burlington, ON, USA). All other solvents and chemicals were of analytical grade.

### 3.2. Obtaining of SP-Rich Extract from the Seaweeds

The seaweeds *Udotea flabellum* (J.Ellis & Solander) M.A. Howe, *Caulerpa cupressoides* (Vahl) C. Agardh, *Caulerpa racemosa* (Forsskål) J. Agardh, *Caulerpa prolifera* (Forsskål) J.V. Lamouroux, *Caulerpa sertulariodes* (S.G. Gmelin) M.A. Howe, *Codium isthmocladum* Vickers, *Ulva lactuca* Linnaeus, *Lobophora variegata* (J.V. Lamouroux) Womersley ex E.C. Oliveira, *Sargassum vulgare* C. Agardh, *Padina gymnospora* (Kützing) Sonder, *Spatoglossum schröederi* (C. Agardh) Kützing, *Dictyota mertensii* (Martius) Kützing, *Dictyota menstrualis* (Hoyt) Schnetter, Hörning & Weber-Peukert, *Canistrocarpus cervicornis* (Kützing) De Paula & De Clerck, *Dictyota ciliolata* Sonder ex Kützing, *Dictyopteris delicatula* J.V. Lamouroux, *Acanthophora spicifera* (M.Vahl) Børgesen, and *Amansia multifida* J.V. Lamouroux, were collected from Búzios Beach, Nísia Floresta-RN, Brazil (05°58′23″ S, 35°04′97″ W). The seaweed *Gracilaria birdiae* E.M. Plastino & E.C. Oliveira, *Sargassum filipendula* C. Agardh, and *Dictyopteris justii* J.V. Lamouroux were collected from Rio do Fogo Beach, Rio do Fogo-RN, Brazil (05°16′22″ S, 35°22′58″ W). The seaweed *Gracilaria caudata* J. Agardh was collected from Camapum beach, Macau-RN, Brazil (05°06′54″ S, 36°38′02″ W). The seaweeds were stored in our laboratory and dried at 50 °C under ventilation in an oven, ground in a blender and incubated with ethanol to eliminate lipids and pigments. About 90 g of powdered seaweed was suspended with 5 volumes of 0.25 M NaCl and the pH was adjusted to 8.0 with NaOH. Next, 900 mg of Prolav 750 (Prozyn Biosolutions, São Paulo, SP, Brazil), a mixture of alkaline proteases, was added for proteolytic digestion. After incubating for 24 h at 60 °C under agitation and periodic pH adjustments, the mixture was filtered through cheesecloth. The filtrates were vacuum dried, resuspended in distilled water, and analyzed.

### 3.3. Fractionation of SP-Rich Extract from U. flabellum

The SP-rich extract (2 L) was fractionated by precipitation with acetone as follows: 0.3 volumes of ice-cold acetone were added to the solution under gentle agitation, and the mixture was maintained at 4 °C for 24 h. The precipitate formed was collected by centrifugation (10,000× *g*, 20 min), vacuum dried, resuspended in distilled water, and analyzed. The process was repeated by adding 0.5, 0.6, 0.7, 1.0, and 2.0 volumes of acetone to the supernatant.

### 3.4. Fractionation of UF-0.5

The UF-0.5 polysaccharides were separated into 2 fractions using a 100 kDa centrifugal filter (Amicon^®^ EMD Millipore, Darmstadt, Germany). The retained fraction contained a 130 kDa sulfated galactan F-II. In addition, the filtered fraction was sub-fractionated using 10, 30, and 50 kDa centrifugal filters (Amicon^®^ EMD Millipore, Darmstadt, Germany) to separate different molecules according to their molecular weights. The presence of SPs was detected only in the retained fraction from the 50 kDa centrifugal filter using the phenol-sulfuric method as described earlier [42].

### 3.5. Gel Permeation Chromatography and Determination Molecular Weight

F-I and F-II were subjected to gel permeation chromatography on a Sephadex G-100 system (140 × 1 cm) (Sigma-Aldrich Co., St. Louis, MO, USA) using 0.2 M acetic acid/0.15 M NaCl as an eluent. The elution was monitored for total sugar and metachromasia as described earlier [19]. A set of dextran standards was used to construct the standard curve for determination of molecular weights of the polysaccharides.

### 3.6. Chemical Analyses and Monosaccharide Composition

Sulfate content was determined by the gelatin-barium method as previously described [38], after acid hydrolysis of the polysaccharides (4 M HCl, 100 °C, 6 h) and using sodium sulfate (1 mg/mL) as the standard. Protein content was measured using Spector’s method [42], wherein the polysaccharides were hydrolyzed with 0.5, 1, 2, and 4 M HCl for various lengths of time (0.5, 1, 2, and 4 h) at 100 °C. Reducing sugars were determined using the Somogyi-Nelson method [17]. After acid hydrolysis, the sugar composition was determined using a LaChrom Elite^®^ HPLC system manufactured by VWR-Hitachi (Tokyo, Japan), equipped with a refractive index detector (RI detector model L-2490 (VWR-Hitachi, Tokyo, Japan). A LichroCART^®^ 250-4 column (250 mm × 40 mm) packed with Lichrospher^®^ 100 NH2 (5 µm) was coupled with the system. The samples weighed 0.2 mg each, and analysis time was 25 min. The following sugars were analyzed as references: arabinose, fructose, fucose, galactose, glucose, glucosamine, glucuronic acid, mannose, and xylose.

### 3.7. APTT Test

All activated partial thromboplastin time (APTT) coagulation assays were performed using a coagulometer as previously described [43] and measured using citrate-treated normal human plasma. All the assays were performed in duplicate and repeated at least 3 times on different days (*n* = 6).

### 3.8. AT-Mediated Thrombin Inhibition Assay

The thrombin inhibition assay was performed on a 96-well microplate, according to the instructions of ACTICHROME^®^ heparin-anti-FIIa Kit (Sekisui Diagnostic, VWR, Radnor, PA, USA). Briefly, 50 μL of AT was incubated at 37 °C for 2 min in the presence of increasing concentrations of heparin or SPs from *U. flabellum*, previously diluted in citrated fresh human plasma. Next, 50 μL of bovine thrombin was added to each well, and the mixture was homogenized and incubated at 37 °C for exactly 2 min. This was followed by addition of 50 μL of the chromogenic substrate. The mixture was homogenized and incubated at 37 °C for 2 min. Following incubation, 80 μL of 30% acetic acid was added to stop the reaction and absorbance was measured against a corresponding blank at 405 nm.

### 3.9. HCII-Mediated Thrombin Inhibition Assay

The HCII-mediated thrombin inhibition test in the presence of SP was performed in a 96-well microplate, with final volume of 100 μL, according to Brito et al. [40]. Briefly, 25 μL of the purified SP in 0.02 M Tris/HCl buffer, 0.15 M NaCl (pH 7.4), and 25 μL HCI (70 nM) were incubated for 2 min at 37 °C. This was followed by addition of 25 μL thrombin (48 nM) and incubation for 1 min at 37 °C. In the next step, 25 μL of the chromogenic substrate, N-benzoyl-Phe-Val-Arg-p-nitroamylidehydrochloride (100 mM), was added, and the mixture was incubated further for 1 min at 37 °C. Finally, 25 μL of 30% acetic acid was added to stop the reaction. The absorbance was recorded at 405 nm. The blank comprised a solution with the same reagents, except for 30% acetic acid, which was added only to stop the reaction. To evaluate the total activity of the enzyme (100% enzymatic activity), the test sample was not added.

### 3.10. Direct Thrombin Inhibition Assay

Direct inhibition of thrombin by SP was determined using the method described above, with the exception that HCII was replaced by 0.02 M Tris/HCl buffer, 0.15 M NaCl (pH 7.4).

### 3.11. B16-F10 Cells

B16-F10 (ATCC^®^ CCL-6475™) is a murine melanoma cell line from a C57BL/6J mouse. These cells were grown in DMEM (Invitrogen, Carlsbad, CA, USA) supplemented with 10% fetal bovine serum/FBS (Cutilab, Campinas-SP, Brazil), penicillin (100 U/mL), and streptomycin (0.1 mg/mL) (Sigma-Aldrich, St Louis, MO, USA), at 37 °C under 5.0% CO_2_.

### 3.12. 3-(4,5-Dimethylthiazol-2-yl)-2,5-diphenyl-tetrazolium bromide (MTT) Test

For MTT assay, cells were seeded into 96-well plates at a density of 0.5 × 10^4^ cells/well and allowed to attach overnight in 300 μL of fresh medium, at 37 °C under 5% CO_2_. In some cases, the cells were added to fibronectin, laminin, or collagen coated wells.

The cells were, then, incubated for 24 h at 37 °C under 5% CO_2_, in the presence of different concentrations (0.01, 0.5, 0.75, and 1 mg/mL) of F-I or F-II. After incubation, traces of sulfated polysaccharide fractions were removed by washing the cells twice with 200 μL PBS. This was followed by addition of 100 μL of fresh medium, as well as 10 μL of 12 mM 3-(4,5-Dimethylthiazol-2-yl)-2,5-diphenyltetrazolium bromide (MTT) dissolved in serum-free medium, to determine the effect of algal sulfated polysaccharides on cell viability. Cells were then incubated for 4 h at 37 °C under 5% CO_2_. The cell capacity to reduce MTT was determined by the colorimetric test of MTT, as previously described [17]. The medium was removed, and the formazan crystals were dissolved with 100 µL of 95% ethanol. After 15 min of shaking in a rocking shaker, absorbance was recorded (570 nm) in a microplate spectrophotometer (Biotek, Winooski, VT, USA). As a negative control, cells were cultured only in Dulbecco Modified Eagle Medium (DMEM) with 10% FBS. Results were expressed as percentage MTT reduction, calculated using Equation (1).

Percentage MTT Reduction = (Absorbance of sample/Absorbance of control) × 100(1)

### 3.13. 5-Bromo-2-Deoxyuridine (BrdU) Incorporation

The cells (5 × 10^3^ cells/well) were seeded into 96-well plates with 300 μL of fresh medium and incubated for 12 h at 37 °C under 5.0% CO_2_. After 12 h, the medium was removed, the samples in DMEM were adjusted to a final concentration between 0.1 and 1.0 mg/mL, and plates were incubated for 24 h at 37 °C under 5.0% CO_2_. After incubation, the unbound samples were removed by washing the cells twice with 200 μL PBS. BrdU incorporation was determined according to manufacturer’s instructions (BrdU cell proliferation assay kit-Cell Signaling, Danvers, MA, USA).

### 3.14. Adhesion Assay

B16-F10 cells (2 × 10^4^) were added to FN-coated wells and allowed to attach for 1 h at 37 °C under 5.0% CO_2_ in the absence (control) or presence of samples (final concentration ranging from 0.1 mg to 1.0 mg/mL), following which, the adhesion assay was performed as previously described [44].

Non-adherent cells in the adhesion assay were evaluated by trypan blue exclusion assay. After incubation for 1 h at 37 °C under 5.0% CO_2_, the culture medium containing the non-adherent cells was collected from each well. Subsequently, it was centrifuged, and the supernatant was discarded. The cell pellet was suspended in serum-free medium (0.1 mL) and 20 µL of this cell suspension was mixed with 20 µL of 0.4% trypan blue (Sigma-Aldrich Co., St. Louis, MO, USA). After ~3 min at room temperature, 20 µL of the trypan blue/cell mixture was introduced into a hemacytometer, and the unstained (viable) and stained (nonviable) cells were separately counted.

### 3.15. Motility Assay

The motility assay was conducted as previously described [45]. Briefly, the undersurface of the polycarbonate membranes of transwell motility chambers (8-µm pore size) (Costar Corp., Richmond, VA, USA) were coated with fibronectin, collagen, or laminin (10 µg/mL in PBS) for 40 min and blocked with 1% BSA (1 h, 37 °C). Cells (5 × 10^5^) in 100 µL of DMEM were added to the upper chambers in the presence or absence (control) of different concentrations of samples. The chambers were maintained at culture conditions (humid atmosphere at 37 °C and 5.0% CO_2_) for 5 h. The cells that migrated were fixed with 2% formaldehyde for 10 min and stained with 1% toluidine blue in borax for an additional 10 min. The number of migrated cells was determined using an inverted microscope. Ten fields of cells were counted for each group (treatment and control) and their values were averaged. Inhibition of cell migration was calculated in percentage terms using the equation:

I% = [1 − (the number of migrated cells in treatment/the number of migrated cells in control)] × 100.

### 3.16. Unspecific Sulfated Polysaccharide Desulfation

Polysaccharide desulfation was performed by solvolysis in dimethyl sulfoxide, as used previously for desulfation of sulfated polysaccharides [38]. The duration of reaction was 1 h. Two desulfated SP were obtained, F-I desulfated and F-II desulfated, containing 7.0% and 7.5% of sulfate groups, respectively.

### 3.17. Statistical Analysis

All data are expressed as mean ± standard deviation of 3 observations (*n* = 3). Statistical analysis was done using one-way ANOVA, followed by the Turkey-Kramer test. All the tests were conducted in SigmaPlot^®^ (Systat software, San Jose, CA, USA). In all cases, statistical significance was set at *p* < 0.05.

## 4. Conclusions

We obtained 15 anticoagulant sulfated polysaccharide-rich extracts from tropical seaweeds. Among these, *Udotea flabellum* extract was the most potent. Two sulfated homogalactans, F-I (130 kDa) and F-II (75 kDa), with anticoagulant activity were isolated from this extract through several bio-guided purification steps. Although F-II had a higher content of sulfate groups than F-I, the activities of both polysaccharides were similar across all tests. The anticoagulant activity of these polysaccharides depends mainly on their ability to potentiate the AT inhibitory action. F-I and F-II also inhibited B16-F10 cell adhesion, migration, and proliferation on a fibronectin-coated surface, but not on laminin- or collagen I-coated surfaces. The anti-proliferative activity of F-I and F-II was dependent on their degree of sulfation. Taken together, the results provide strong evidence for the potential of these sulfated galactans isolated from *U. flabellum*, making these compounds an interesting option for future investigations that aim to design new anticoagulant/antitumor agents.

## Figures and Tables

**Table 1:** Anticoagulant activity of SP-rich extracts obtained from several tropical seaweeds.

	APTT
Green Seaweeds	µg *
*Caulerpa cupressoides*	7.0 ± 0.8
*Caulerpa sertulariodes*	6.0 ± 0.8
*Caulerpa prolifera*	8.0 ± 0.7
*Caulerpa racemosa*	8.0 ± 0.5
*Codium isthmocladum*	10.0 ± 0.7
*Udotea flabellum*	3.0 ± 0.3
*Ulva lactuca*	10.0 ± 0.9
Brown seaweeds
*Lobophora variegata*	25.0 ± 1.5
*Sargassum vulgare*	20.0 ± 1.9
*Padina gymnospora*	50.0 ± 2.5
*Sargassum felipendula*	nd
*Spatglossum schröederi*	nd
*Dictyota mertensii*	35.0 ± 4.7
*Dictyopteris delicatula*	45.0 ± 1.8
*Dictyota menstrualis*	nd
*Canistrocarpus cervicornis*	15.0 ± 0.9
*Dictyopteris justii*	nd
*Dictyota ciliolata*	nd
Red seaweeds	
*Gracilaria birdiae*	100.0 ± 5.0
*Gracilaria caudata*	nd
*Amansia. multifida*	nd
*Achantophora especifera*	nd
Heparin	1.0

* Data are reported as amount (µg) required to double APTT compared with saline control. APTT = activated partial thromboplastin time; nd = anticoagulant activity not detected until 200 µg.

**Table 2:** Yield and anticoagulant activity of SP-rich fractions extracted from the *U. flabellum*.

*Udotea* Fractions	Yield ^a^ (%)	APTT ^b^ (µg)
UF-0.3	5.8	20.0
UF-0.5	33.8	2.5
UF-0.6	10.3	2.5
UF-0.7	14.1	5.0
UF-1.0	14.8	20.0
UF-2.0	21.2	nd

^a^ All polysaccharides obtained by acetone precipitation were dried and weighed, and the total mass of each sample corresponded to 100%. ^b^ Data are reported as amount (µg) required to double APTT compared with saline control. APTT = activated partial thromboplastin time; nd = anticoagulant activity not detected until 200 µg.

**Table 3:** Chemical composition and anticoagulant activity of polysaccharides extracted from *U. flabellum*.

SPs	Yield (%) ^a^	Sugar (%)	Sulfate (%)	Protein (%)	APTT ^b^ (µg)		Molar Ratio		
						Gal	Glu	Man	Xyl
URSF		75.2 ± 2.1	18.2 ± 0.5	5.0 ± 1.8	3.0	1.0	1.2	0.5	0.3
UF-0.5		82.0 ± 0.9	18.7 ± 0.8	0.6 ± 0.3	2.5	1.0	0.5	-	-
F-I	62.2 ± 2.6	84.0 ± 1.6	14.0 ± 1.1	nd	1.0	1.0	-	nd	nd
F-II	25.1 ± 1.3	76.0 ± 2.8	24.0 ± 0.5	nd	1.0	1.0	-	nd	nd
F-III	7.0 ± 0.6	72.2 ± 1.8	2.0 ± 0.3	8.0 ± 0.7	5.0	1.0	1.0	0.1	-
F-IV	3.1 ± 0.3	58.2 ± 3.5	3.0 ± 0.8	14 ± 1.3	1.0	1.0	1.0	0.1	0.2
F-V	3.0 ± 0.2	66.5 ± 1.8	3.0 ± 0.4	17 ± 0.9	5.0	1.0	0.8	1.0	-

^a^ All obtained samples were dried and weighed, and the total mass corresponded to 100%. ^b^ Data are reported as amount (µg) required to double APTT compared with saline control. Heparin was required in a quantity of 1.0 µg to double APTT compared with saline control. Gal: galactose; Xyl: xylose; Man: mannose; Gluc: glucose; -: Traces; nd—not detected. USRF—Udotea SP-rich fraction.

**Table 4:** Anticoagulant, anti-proliferative, anti-adhesive, and anti-migratory activities of desulfated F-I and F-II.

Property	Desulfated F-I	Desulfated F-II
Anticoagulant	ND until 200 µg	ND until 200 µg
Anti-proliferative	ND until 1.0 mg/mL	ND until 1.0 mg/mL
Anti-adhesion when FN was substrate	ND until 1.0 mg/mL	ND until 1.0 mg/mL
Anti-migratory when FN was substrate	ND until 1.0 mg/mL	ND until 1.0 mg/mL
Anti-proliferative when FN was substrate	~40% at 1.0 mg/mL	~40% at 1.0 mg/mL

## References

[1] The Top 10 Causes of Death. http://www.who.int/mediacentre/factsheets/fs310/en/.

[2] Elyamany G., Alzahrani A.M., Bukhary E. (2014). Cancer-associated thrombosis: An overview. Clin. Med. Insights Oncol..

[3] Ikushima S., Ono R., Fukuda K., Sakayori M., Awano N., Kondo K. (2016). Trousseau’s syndrome: Cancer-associated thrombosis. Jpn. J. Clin. Oncol..

[4] Mackman N. (2004). Role of tissue factor in hemostasis, thrombosis, and vascular development. Arterioscler Thromb. Vasc. Biol..

[5] Schmaier A.A., Ambesh P., Campia U. (2018). Venous thromboembolism and cancer. Curr. Cardiol. Rep..

[6] Warkentin T.E., Cook R.J., Sarode R., Sloane D.A., Crowther M.A. (2015). Warfarin-induced venous limb ischemia/gangrene complicating cancer: A novel and clinically distinct syndrome. Blood.

[7] Rosenbaum A.N., Yu R.C., Rooke T.W., Heit J.A. (2014). Venous gangrene and intravascular coagulation and fibrinolysis in a patient treated with rivaroxaban. Am. J. Med..

[8] Nader H.B., Lopes C.C., Rocha H.A.O., Santos E.A., Dietrich C.P. (2004). Heparins and heparinoids: Occurrence, structure and mechanism of antithrombotic and hemorrhagic activities. Curr. Pharm. Des..

[9] Noble S. (2014). Heparins and cancer survival: Where do we stand?. Thromb. Res..

[10] Nahain A.A., Ignjatovic V., Monagle P., Tsanaktsidis J., Ferro V. (2018). Heparin mimetics with anticoagulant activity. Med. Res. Rev..

[11] Lima M.A., Rudd T.R., de Farias E.H., Ebner L.F., Gesteira T.F., de Souza L.M., Mendes A., Córdula C.R., Martins J.R.M., Hoppensteadt D. (2011). A new approach for heparin standardization: Combination of scanning UV spectroscopy, nuclear magnetic resonance and principal component analysis. PLoS ONE.

[12] Wang L., Wang X., Wu H., Liu R. (2014). Overview on biological activities and molecular characteristics of sulfated polysaccharides from marine green algae in recent years. Mar. Drugs.

[13] Jiao G., Yu G., Zhang J., Ewart H.S. (2011). Chemical structures and bioactivities of sulfated polysaccharides from marine algae. Mar. Drugs.

[14] Vasconcelos A.A., Pomin V.H. (2018). Marine carbohydrate-based compounds with medicinal properties. Mar. Drugs.

[15] Arata P.X., Quintana I., Canelón D.J., Vera B.E., Compagnone R.S., Ciancia M. (2015). Chemical structure and anticoagulant activity of highly pyruvylated sulfated galactans from tropical green seaweeds of the order Bryopsidales. Carbohydr. Polym..

[16] Siddhanta A.K., Shanmugam M., Mody K.H., Goswami A.M., Ramavat B.K. (1999). Sulphated polysaccharides of *Codium dwarkense* Boergs. from the west coast of India: Chemical composition and blood anticoagulant activity. Int. Biol. Macromol..

[17] Presa F.B., Marques M.L.M., Viana R.L.S., Nobre L.T.D.B., Costa L.S., Rocha H.A.O. (2018). The protective role of sulfated polysaccharides from green seaweed *Udotea flabellum* in cells exposed to oxidative damage. Mar. Drugs.

[18] Farias E.H.C., Pomin V.H., Valente A.-P., Nader H.B., Rocha H.A.O., Mourão P.A.S. (2008). A preponderantly 4-sulfated, 3-linked galactan from the green alga *Codium isthmocladum*. Glycobiology.

[19] Costa M.S.S.P., Costa L.S., Cordeiro S.L., Almeida-Lima J., Dantas-Santos N., Magalhães K.D., Sabry D.A., Albuquerque I.R.L., Pereira M.R., Leite E.L. (2012). Evaluating the possible anticoagulant and antioxidant effects of sulfated polysaccharides from the tropical green alga *Caulerpa cupressoides* var. flabellata. J. Appl. Phycol..

[20] Percival E., McDowell R.H., Tanner W., Loewus F.A. (1981). Algal walls: Composition and biosynthesis. Encyclopedia of Plant Physiology.

[21] Bilan M.I., Vinogradova E.V., Shashkov A.S., Usov A.I. (2007). Structure of a highly pyruvylated galactan sulfate from the pacific green alga *Codium yezoense* (Bryopsidales, Chlorophyta). Carbohydr. Res..

[22] Ciancia M., Alberghina J., Arata P.X., Benavides H., Leliaert F., Verbruggen H., Estevez J.M. (2012). Characterization of cell wall polysaccharides of the coencocytic green seaweed *Bryopsis plumosa* (Bryopsidaceae, Chlorophyta) from the Argentine coast. J. Phycol..

[23] Ngo D.-H., Kim S.-K. (2013). Sulfated polysaccharides as bioactive agents from marine algae. Int. J. Biol. Macromol..

[24] Hayakawa Y., Hayashi T., Lee J., Srisomporn P., Maeda M., Ozawa T., Sakuragawa N. (2000). Inhibition of thrombin by sulfated polysaccharides isolated from green algae. Biochim. Biophys. Acta.

[25] Mao W., Zang X., Li Y., Zhang H. (2006). Sulfated polysaccharides from marine green algae *Ulva conglobata* and their anticoagulant activity. J. Appl. Phycol..

[26] Hisada Y., Geddings J.E., Ay C., Mackman N. (2015). Venous thrombosis and cancer: From mouse models to clinical trials. J. Thromb. Haemost..

[27] Lucas A., Yaron J.R., Zhang L., Ambadapadi S. (2018). Overview of Serpins and Their Roles in Biological Systems. Methods Mol. Biol..

[28] Lokwani R., Azmi N.S., Yusoff M.M., Ichwan S.J.A. (2014). Beyond anticoagulant: Heparin as a potential anti-cancer agent. J. Biochem. Microbiol. Biotechnol..

[29] Niers T.M., Brüggemann L.W., Klerk C.P., Muller F.J., Buckle T., Reitsma P.H., Richel J., Spek C.A., van Tellingen O., van Noorden C.J. (2009). Differential effects of anticoagulants on tumor development of mouse cancer cell lines B16, K1735 and CT26 in lung. Clin. Exp. Metastasis.

[30] Dasari S., Tchounwou P.B. (2014). Cisplatin in cancer therapy: Molecular mechanisms of action. Eur. J. Pharmacol..

[31] Sonbol H.S. (2018). Extracellular Matrix Remodeling in Human Disease. J. Microsc. Ultrastruct..

[32] Rocha H.A.O., Franco C.R.C., Trindade E.S., Veiga S.S., Leite E.L., Dietrich C.P., Nader H.B. (2005). Fucan inhibits Chinese hamster ovary cell (CHO) adhesion to fibronectin to extracellular matrix. Planta Med..

[33] Trindade E.S., Constance O., Jamur M.C., Rocha H.A.O., Franco C.R.C., Bouças R.I., Jarrouge T.R., Pinhal M.A.S., Tersariol I.L.S., Gouvêa T.C. (2008). The binding of heparin to the extracellular matrix of endothelial cells up-regulates the synthesis of an antithrombotic heparan sulfate proteoglycan. J. Cell Physiol..

[34] Lahaye M., Robic A. (2007). Structure and functional properties of ulvan, a polysaccharide from green seaweeds. Biomacromolecules.

[35] Raimondi L., Banchelli G., Dalmazzi D., Mulinacci N., Romani A., Vincieri F.F., Pirisino R. (2000). *Sedum telephium* L. polysaccharide content affects MRC5 cell adhesion to laminin and fibronectin. J. Pharm. Pharmacol..

[36] Liu J.M., Bignon J., Haroun-Bouhedja F., Bittoun P., Vassy J., Fermandjian S., Wdzieczak-Bakala J., Boisson-Vidal C. (2005). Inhibitory effect of fucoidan on the adhesion of adenocarcinoma cells to fibronectin. Anticancer Res..

[37] Hanahan D., Weinberg R.A. (2011). Hallmarks of cancer: The next generation. Cell.

[38] Nobre L.T., Vidal A.A.J., Almeida-Lima J., Oliveira R.M., Paredes-Gamero E.J., Medeiros V.P., Trindade E.S., Franco C.R.C., Nader H.B., Rocha H.A.O. (2013). Fucan effect on CHO cell proliferation and migration. Carbohydr. Polym..

[39] Haroun-Bouhedja F., Lindenmeyer F., Lu H., Soria C., Jozefonvicz J., Boisson-Vidal C. (2002). In vitro effects of fucans on MDA-MB231 tumor cell adhesion and invasion. Anticancer Res..

[40] Brito A.S., Cavalcante R.S., Cavalheiro P.V., Palhares L.C.G.F., Nobre L.T.D.B., Andrade G.P.V., Nader H.B., Lima M.A., Chavante S.F. (2018). Anti-IIa activity and antitumor properties of a hybrid heparin/heparan sulfate-like compound from *Litopenaeus vannamei*. Int. J. Biol. Macromol..

[41] Biscaia S.M.P., Carbonero E.R., Bellan D.L., Borges B.S., Costa C.R., Rossi G.R., Trindade E.S. (2017). Safe therapeutics of murine melanoma model using a novel antineoplasic, the partially methylated mannogalactan from *Pleurotus eryngii*. Carbohydr. Polym..

[42] Galinari E., Almeida-Lima J., Macedo G.R., Mantovani H.C., Rocha H.A.O. (2018). Antioxidant, antiproliferative, and immunostimulatory effects of cellwall-D-mannan fractions from *Kluyveromyces marxianu*. Int. J. Biol. Macromol..

[43] Costa L.S., Fidelis G.P., Cordeiro S.L., Oliveira R.M., Sabry D.A., Câmara R.B., Nobre L.T., Costa M.S., Almeida-Lima J., Farias E.H. (2010). Biological activities of sulfated polysaccharides from tropical seaweeds. Biomed. Pharmacother..

[44] Rocha H.A.O., Franco C.R.C., Trindade E.S., Carvalho L.C.M., Veiga S.S., Leite E.L., Dietrich C.P., Nader H.B. (2001). A fucan from the brown seaweed *Spatoglossum schröederi* inhibits Chinese hamster ovary cell adhesion to several extracelular matrix proteins. Braz. J. Med. Biol. Res..

[45] Franco C.R.C., Trindade E.S., Rocha H.A.O., Silveira R.B., Paludo K.S., Chammas R., Veiga S.S., Nader H.B., Dietrich C.P. (2009). Glycosaminoglycan chains from alpha5beta1 integrin are involved in fibronectin-dependent cell migration. Biochem. Cell Biol..

